# Lipid Alterations in Chronic Nonspecific Low Back Pain in the Chinese Population: A Metabolomic and Lipidomic Study

**DOI:** 10.3390/bioengineering11111114

**Published:** 2024-11-05

**Authors:** Wen Tang, Hong-Jiang Wang, Su-Ying Luo, Si-Yun Zhang, Hao Xie, Hua-Qing Chen, Chu-Huai Wang, Zhou Zhang

**Affiliations:** 1 Department of Rehabilitation Medicine, The First Affiliated Hospital, Sun Yat-sen University, Guangzhou 510275, China; tangw36@mail.sysu.edu.cn (W.T.); wanghj73@mail.sysu.edu.cn (H.-J.W.); luosy@mail.sysu.edu.cn (S.-Y.L.); zhangsy63@mail.sysu.edu.cn (S.-Y.Z.); xieh66@mail.sysu.edu.cn (H.X.); chenhuaqingdoc@163.com (H.-Q.C.); 2 Department of Rehabilitation, Tongji Hospital, Tongji Medical College, Huazhong University of Science and Technology, Wuhan 430030, China

**Keywords:** chronic nonspecific low back pain, metabolomic, lipidomic, monoacylglycerol, diacylglycerol, triacylglycerol, phosphatidylserine

## Abstract

Chronic nonspecific low back pain (cNLBP) accounts for approximately 90% of low back pain cases, affecting 65–80% of the population and significantly impacting life quality and productivity. This condition also leads to substantial financial burden. Although there have been advancements, a comprehensive understanding of the underlying etiology of cNLBP remains elusive, resulting in less than optimal treatment outcomes. This study aimed to examine the correlation between lipid variations and the development of cNLBP. The cohort consisted of 26 healthy volunteers (HV group) and 30 cNLBP patients, with an assessment of metabolites and lipid composition in both groups. Metabolomic results revealed significant alterations in lipid-associated metabolites between the HV and cNLBP groups. Subsequent lipid analysis revealed that monoacylglycerols (MAGs) increased approximately 1.2-fold (*p* = 0.016), diacylglycerols (DAGs) increased approximately 1.4-fold (*p* = 0.0003), and phosphatidylserine (PS) increased approximately 1.4-fold (*p* = 0.011). In contrast, triacylglycerol (TAG) decreased to about 0.7-fold (*p* = 0.035) in the cNLBP group compared to the HV group. The contrasting trends in MAG/DAG and TAG levels indicated that the imbalance between MAG/DAG and TAG may have an impact on the development of cNLBP. This study has provided new insights into the relationship between the progression of cNLBP and specific lipids, suggesting that these lipids could serve as therapeutic targets for cNLBP.

## 1. Introduction

Low back pain (LBP), stretching from the 12th rib to the hip crease and occasionally to the upper thigh, resembles musculoskeletal discomfort rather than a disease, akin to headaches and chest pain [1]. There are two main categories of LBP: specific and nonspecific. Specific LBP is causally linked to infection, trauma, injury, or structural aberration and thereby has a clear underlying etiology. Conversely, nonspecific LBP lacks a precise cause or structural justification and makes up around 90% of LBP cases [2]. LBP is also typically categorized based on its duration, with categories including acute, subacute, and chronic LBP [3].

Chronic nonspecific low back pain (cNLBP) is a common chronic condition characterized by pain and impaired movement, which can negatively impact the working capacity of both young and middle-aged individuals [4]. With reduced physical activity and work absenteeism, cNLBP ranks just behind upper respiratory diseases in terms of global impact on quality of life. cNLBP can pose a significant financial burden on people, households, societies, businesses, and governments [5,6]. Specifically, a 2010 study on the Global Burden of Disease pinpointed cNLBP as a major contributor to disability and absenteeism from work, underscoring the importance of effective cNLBP management [7]. A study on the epidemiology of cNLBP in China found that the incidence of cNLBP among Chinese residents is approximately 30%. Most patients with cNLBP are aged between 20 and 49 years, with the condition typically lasting 1–5 years along with significant impacts on sleep, mental health, and overall well-being [8]. Extensive research has been devoted to understanding the complexities of cNLBP from physiological, psychosocial, and behavioral perspectives, aiming to improve both prevention strategies and disease management [9]. Despite progress, a comprehensive understanding of the underlying etiology of cNLBP remains elusive, resulting in suboptimal treatment outcomes.

In recent years, omics technology has become increasingly prevalent in medical and biological research [10]. A myriad of omics tools has allowed for systematic analysis of various samples, enabling integrated cross-group comparisons to unravel the complexity of the innate biological systems. This, in turn, has transformed our understanding of disease development and treatment [11,12]. Among the many omics technologies available to date, metabolomics aims to utilize qualitative and quantitative analysis of endogenous small-molecule metabolites within biological samples. The detection of metabolite levels can provide valuable information on normal and altered metabolic pathways. Such information can be crucial in gaining insights into disease diagnoses and treatment [13]. In addition, individual metabolite level changes can provide valuable insights for monitoring various diseases and improving personalized medical care [14]. Currently, metabolomics studies have been carried out for various conditions, such as fibromyalgia, chronic widespread pain, musculoskeletal pain, neuropathic pain, and pelvic pain [15,16,17,18]. As an essential class of cellular metabolites, lipids play indispensable roles in various immune responses and disease progression. Lipidomic is now being widely employed in studies related to chronic pain conditions, including but not limited to inflammatory pain, fibromyalgia, and chronic arthralgia [19,20,21]. However, whether the pathogenesis of cNLBP is associated with underlying lipidomic changes remains elusive.

In this study, we compared the metabolomic profiles of patients with cNLBP versus matched healthy individuals with a focus on lipid-related metabolites. A comprehensive analysis of serum lipidome revealed that the abundance of 5 monacylglycerol (MAG) components, 13 diacylglycerol (DAG) components, and 10 phosphatidylserine (PS) components was significantly increased in the cNLBP group compared to the HV group. Conversely, an analysis of 411 triacylglycerols (TAGs) revealed that approximately 10% exhibited a notable decrease in abundance in cNLBP patients compared to the HV group. By integrating metabolomic and lipidomic analyses, this study offers original insights into the potential role of glycerophospholipids, among other lipid types, as a pathogenic factor in cNLBP.

## 2. Materials and Methods

### 2.1. Participants

Healthy volunteers (HV group) and cNLBP patients (cNLBP group) were recruited for this study through advertisements. Individuals between the ages of 18 and 65 years were included if they had been experiencing pain extending from the lower rib to the inferior gluteal fold for over three months continuously or intermittently for over six months [22]. Confirmation of the cNLBP diagnosis was conducted by two approved medical practitioners utilizing the diagnostic criteria outlined by the American Pain Society and the American College of Physicians [23]. Furthermore, only cNLBP patients with a Visual Analog Scale (VAS) score of at least 2 were included in the study [24]. Other inclusion criteria included being right-hand dominant, absence of neurological diseases or intracranial lesions, and not having received any treatment for back pain within the preceding three months.

Meanwhile, potential participants who did not meet specific health requirements were excluded from the study. Specifically, participants who had undergone recent or ongoing pregnancy, experienced pain radiating from the back, or were suffering from postpartum low back pain or menstrual discomfort were excluded from the study. Furthermore, those with known inflammatory spinal diseases, vertebral fractures, severe osteoporosis, auto-inflammatory arthritis, cancer, or significant unexplained weight loss were also not considered. Individuals with cardio-cerebrovascular conditions or endocrine disorders were not suitable to participate. In addition, those with mental health conditions requiring medication, patients not willing to sign the consent form for the study or comply with the research protocol, and individuals who were having dependencies on alcohol or substances were also excluded from participation in the study.

The intensity of pain in all subjects was evaluated utilizing VAS prior to the study, and blood samples were gathered for metabolomic and lipidomic analysis.

### 2.2. Metabolomic Measurement

Samples for metabolomic analysis were prepared according to the procedure detailed in the study by Wang and colleagues [25]. The process began with the collection of venous blood through heparinization tubes, which was then subjected to centrifugation at 2500 rpm at 4 °C to separate the serum. Following this, 100 μL of serum was added to a 400 μL solution comprising methanol, acetonitrile, and water. The mixture was then sonicated for a duration of 10 min at 4 °C. Following this, the samples were incubated at −20 °C for one hour. After centrifugation, the supernatant was collected and evaporated under vacuum. The samples were then reconstituted with a 1:1 ratio of acetonitrile to water solution.

A metabolite analysis was conducted using a Shimadzu LC-30A (Shimadzu, Japan) connected to a QTRAP 4500 mass spectrometer. The experiment was conducted using the UPLC BEH Amide column. The temperature of the oven remained constant at 55 °C. The injection volume and flow rate of the experiment were set at 5 μL and 0.3 mL/min, respectively. The mobile phase consisted of a combination of reagent A and reagent B. Reagent A contained 100% H_2_O, 0.025 M ammonium acetate, and 0.025 M ammonium hydroxide, while reagent B was made up of 100% acetonitrile. The gradient program was as follows: 0–1 min, 15% A and 85% B; 1–12 min, 35% A and 65% B; 12–12.1 min, 60% A and 40% B; 12.1–15 min, 60% A and 40% B; 15–15.1 min, 15% A and 85% B; and 15.1–20 min, 15% A and 85% B. The voltage of the ion source was adjusted to either −4500 V or 5500 V, while the temperature of the ion source was maintained at 600 °C. The curtain gas pressure was set at 20 psi, with the atomizing and auxiliary gases both configured to 60 psi. Quality control (QC) samples were included in the test every third sample to adjust for drift and verify data quality. After examinations, the raw data were transformed into an mzXML format using ProteoWizard (version 3.0), an internet tool for aligning peaks, correcting retention time, and extracting peak areas through XCMS. With reference to the human metabolome database (HMDB), metabolites were identified by matching metabolite compositions using *m*/*z* details and spectrograms within a 25 ppm range.

### 2.3. Lipidomic Analysis

Lipid samples were prepared according to the procedures outlined by Xuan et al. with certain adjustments [26]. In short, the process began with the collection of venous blood through heparinization tubes, which was then subjected to centrifugation at 2500 rpm at 4 °C to separate the serum. Following this, lipid standards were added into 200 μL of serum, followed by the addition of 400 μL of MTBE and 80 μL of methanol. The mixture was vortexed for 30 s. Following vertexing, samples underwent centrifugation. The top layer was retrieved and transferred to fresh tubes. Under vacuum evaporation, the supernatant was evaporated, followed by reconstitution of each sample in a 1:1 ratio of methylene chloride to methanol.

A lipid analysis was conducted using a Shimadzu LC-30A (Shimadzu, Japan) connected to a QTRAP 6500 mass spectrometer (AB Sciex, Framingham, MA 01701, USA). The experiment was conducted using the UPLC BEH Amide column. The temperature of the oven remained constant at 55 °C. The injection volume and flow rate of the experiment were set at 5 μL and 0.26 mL/min, respectively. Solvent A in the mobile phase consisted of a 40:60 (*v*/*v*) ratio of H_2_O and acetonitrile, supplemented with 0.01 M of ammonium acetate. Solvent B consisted of a 10:90 (*v*/*v*) mixture of acetonitrile and isopropanol, along with 0.01 M of ammonium acetate. The gradient program was as follows: 0–1 min, 68% A and 32% B; 1.5–15.5 min, 15% A and 85% B; 15.5–15.6 min, 3% A and 97% B; 15.6–18 min, 3% A and 97% B; 18–18.1 min, 68% A and 32% B; and 18.1–20 min, 68% A and 32% B. The voltage of the ion source was adjusted to either -4500 V or 5500 V, while the temperature of the ion source was maintained at 600 °C. The curtain gas pressure was set at 20 psi, with atomizing and auxiliary gases both configured to 60 psi. Mass spectrometric data acquisition utilized multiple reaction monitoring (MRM). QC samples were generated using test samples to monitor instrumental drift and assess data integrity during LC-MS analysis.

### 2.4. Statistical Analysis

The abundance of different metabolites and lipids was quantified based on the peak area. The data was normalized with a reference sample and analyzed in accordance with the procedures specified on the site https://www.metaboanalyst.ca/, accessed on 26 September 2024 [27,28,29]. The metabolomic analysis employed a partial least squares-discriminant analysis (PLS-DA) to detect the highest covariance between the HV group and the cNLBP group. For lipidomic analysis, a comparable objective was achieved through using orthogonal partial least-squares discriminant analysis (OPLS-DA). Correlation heatmaps were utilized to evaluate the relationships between lipid molecules, while a hierarchical clustering analysis was used to depict variations in lipid content across samples. Furthermore, a pathway analysis was conducted using the web-based tool METPA.

Prior to group comparisons, the raw data underwent logarithmic transformations and normality testing. Based on MetaboAnalyst 5.0, group differences were visually illustrated through PLS-DA and OPLS-DA. A statistical analysis employed a Student’s *t*-test (*p* < 0.05). Data were presented as means ± SEM.

## 3. Results

### 3.1. Metabolite Alterations Between Healthy Volunteers and cNLBP Patients

We recruited 26 healthy volunteers (HV group) and 30 cNLBP patients (cNLBP group). The flow chart of the study is shown in Supplemental Figure S1. The cNLBP patients experienced significant pain based on the VAS score. Additionally, there were no notable disparities in age, height, weight, or BMI among the participants in both groups. Demographic information is available in Table 1.

Abbreviation: BMI, body mass index; VAS, visual analogue scale.

**Figure 1:**
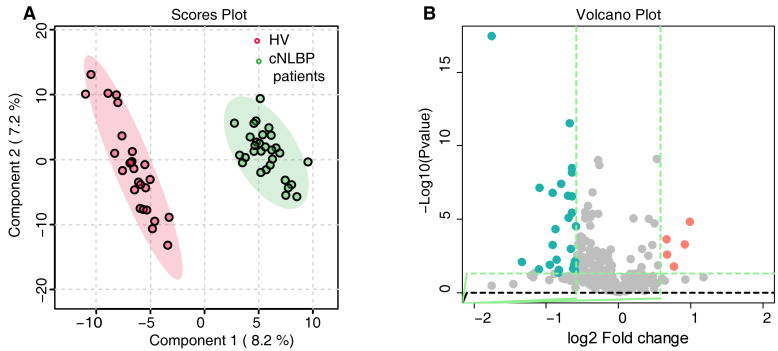
**Metabolomics analysis in the HV and cNLBP groups.** (**A**) PLS-DA score plots were generated comparing HV and cNLBP patients. (**B**) Volcano plot showing differential metabolites between the HV and cNLBP groups.

To investigate potential pathogenic factors of cNLBP, we compared the serum metabolite profiles between the two groups. A PLS-DA analysis was performed to assess the system’s appropriateness. The PLS-DA score plot showed clear differentiation between the HV and cNLBP patients (Figure 1A). A total of 521 metabolites were detected, of which 20 exhibited noteworthy alterations (fold change ≥ 1.5 and *p* < 0.05). Out of the 20 metabolites, 2 metabolites displayed up-regulation, whereas 18 showed down-regulation in the cNLBP group (Figure 1B).

**Figure 2:**
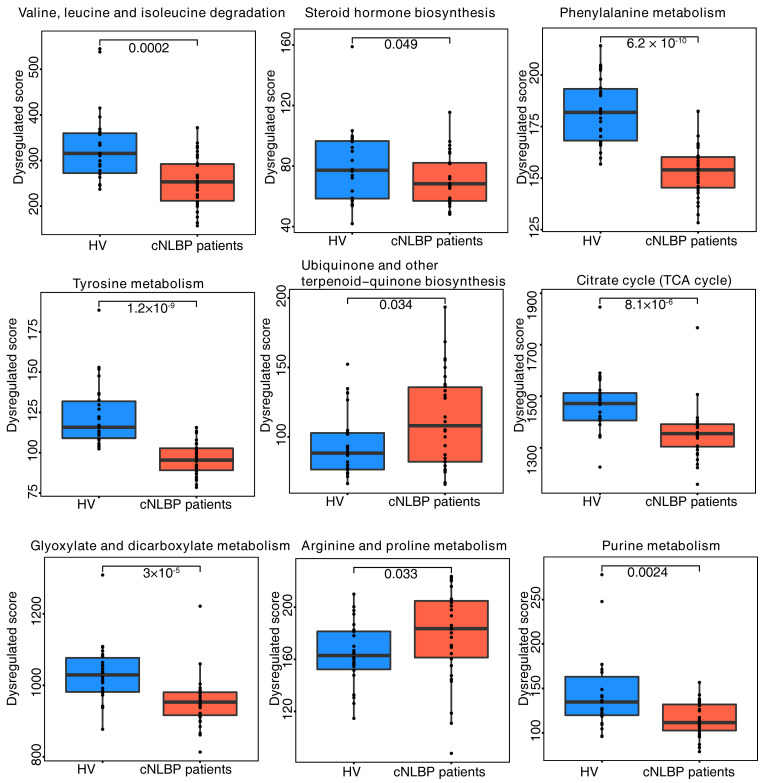
**Pathway enrichment analysis in the HV and cNLBP groups.** Pathway enrichment analysis identified nine distinct metabolic pathways that were significantly enriched in the cNLBP group compared with the HV group (*p*-value cutoff ≤ 0.05).

After comprehensive examinations of the KEGG pathway for differing metabolites, it was noted that nine pathways exhibited notable disparities among the two groups. These pathways include valine, leucine, and isoleucine degradation, tyrosine metabolism, glyoxylate and dicarboxylate metabolism, steroid hormone biosynthesis, ubiquinone and other terpenoid−quinone biosynthesis, arginine and proline metabolism, phenylalanine metabolism, citrate cycle (TCA cycle), and purine metabolism (Figure 2).

**Figure 3:**
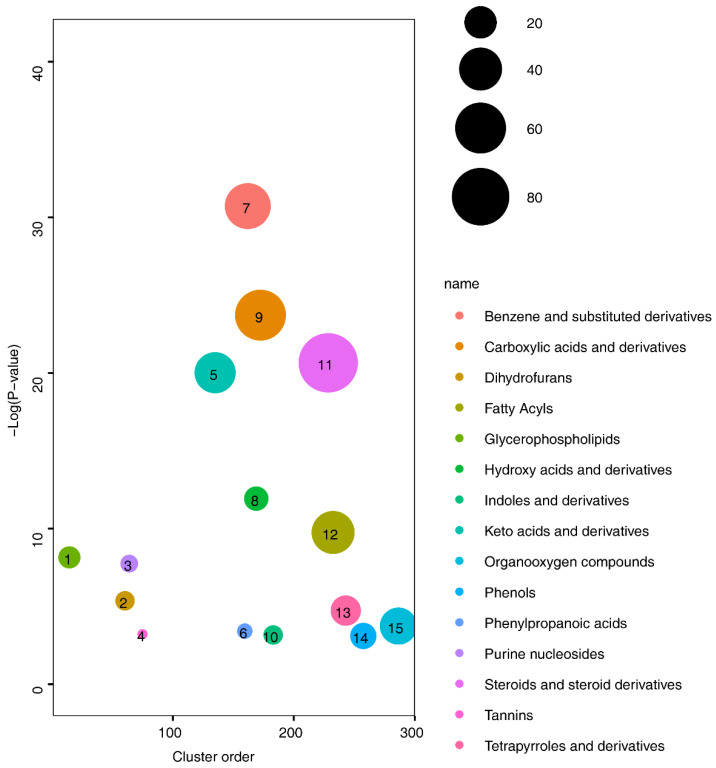
**Class enrichment analysis in the HV and cNLBP groups.** Class enrichment analysis identified 15 types of metabolites that were significantly enriched in the cNLBP group compared with the HV group (*p*-value cutoff ≤ 0.05).

Subsequent clustering of the identified differential metabolites revealed enrichment in 15 categories of substances: benzene and substituted derivatives, carboxylic acids and derivatives, fatty acyls (FAs), glycerophospholipids, hydroxy acids and derivatives, indoles and derivatives, keto acids and derivatives, organooxygen compounds, phenols, phenylpropanoic acids, purine nucleosides, steroids and steroid derivatives, tannins, and tetrapyrroles and derivatives (Figure 3). Among all identified categories, alterations in lipid-associated metabolites, such as fatty acyls, glycerophospholipids, steroids, and steroid derivatives, suggest that changes in lipid composition may be linked to cNLBP. Lipids play crucial roles in various immune responses and pain regulation. However, the specific lipid components involved and whether these changes are directly associated with cNLBP remain unclear. Therefore, we employ lipidomics to investigate the specific alterations in lipid profiles.

### 3.2. Lipid Composition Analysis in Healthy Volunteers and cNLBP Patients

**Figure 4:**
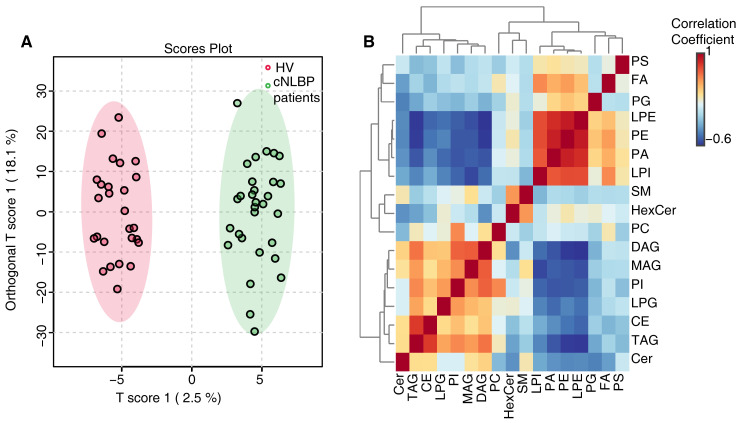
**Lipidome analysis in the HV and cNLBP groups.** (**A**) Orthogonal PLS−DA score plots were generated comparing HV and cNLBP patients. (**B**) Correlation analysis was conducted on the significantly different lipids, with different colors representing the level of Pearson’s correlation coefficient.

**Figure 5:**
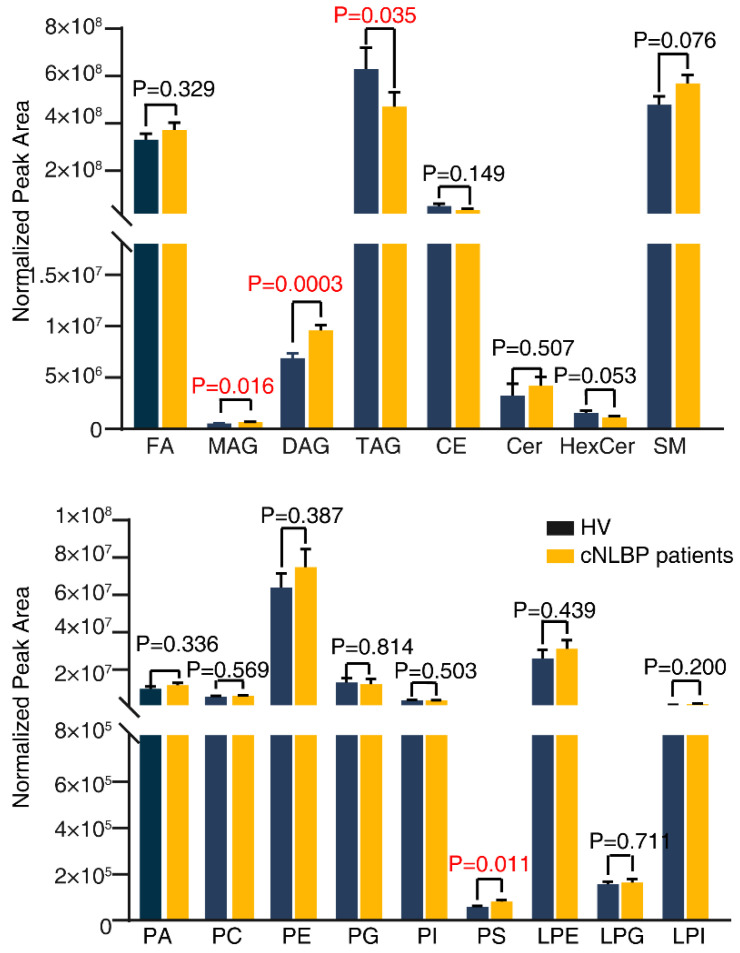
**Identification of changes in lipid components in the HV and cNLBP groups.** Lipid analysis was performed on samples collected from the HV and cNLBP groups. Significant variations in lipid levels compared to HV were identified using Student’s *t*-test. Significant differences were denoted when the *p*-value was less than 0.05.

To further investigate lipid composition differences between HV and cNLBP patients, we conducted a quantitative lipid analysis and identified a total of 1086 lipids, categorized into 17 classes. These classes include FA, MAG, DAG, TAG, CE (cholesterol ester), Cer (ceramide), HexCer (hexose ceramide), SM (sphingomyelin), PA (phosphatidic acid), PC (phosphatidylcholine), PE (phosphatidyl ethanolamine), PG (phosphatidylglycerol), PI (phosphatidyl inositol), PS, LPE (lysophosphatidylethanolamine), LPG (lysophosphatidylglycerol), and LPI (lysophosphatidylinositol). An orthogonal PLS-DA analysis was conducted using the MetaboAnalyst R software package, revealing distinct lipidomic profiles between individuals in the HV and the cNLBP groups (Figure 4A). These findings indicated potential differences in lipid metabolism in individuals with cNLBP compared with healthy controls. Correlation analysis was then conducted on the identified lipids using the Pearson correlation coefficient to examine the level of correlation among different lipids (Figure 4B). Out of the 17 lipid classes analyzed, only 4 exhibited noticeable variations between healthy individuals and cNLBP patients. The remaining 13 lipids did not show any significant alterations. In the cNLBP group, there was a notable rise in MAG, DAG, and PS compared to the HV group, whereas the levels of TAG were significantly reduced (Figure 5).

### 3.3. Altered Glycerolipid Levels in cNLBP Patients

**Figure 6:**
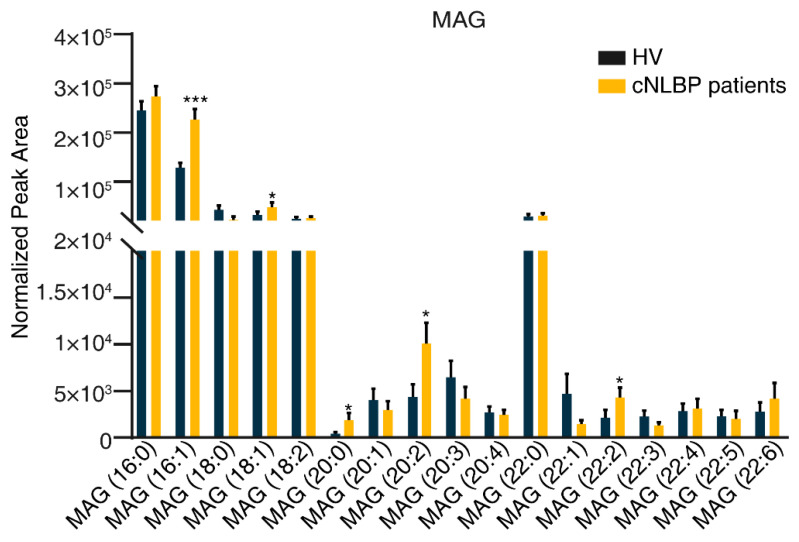
**Detecting significant variations in the constituents of MAG in the HV and cNLBP groups.** To compare the changes in MAG levels between the HV and cNLBP groups, a Student’s *t*-test was performed across the two groups. A *p*-value of less than 0.05 was deemed statistically significant and denoted with an asterisk.

To pinpoint specific lipid components implicated in cNLBP, we delved into the detailed changes within the four types of lipids significantly altered. Out of the 17 MAG components identified, 5 exhibited a significant increase in the patient group: MAG (16:1), MAG (18:1), MAG (20:0), MAG (20:2), and MAG (20:2), while the remaining 12 showed no notable variations between the groups (Figure 6).

**Figure 7:**
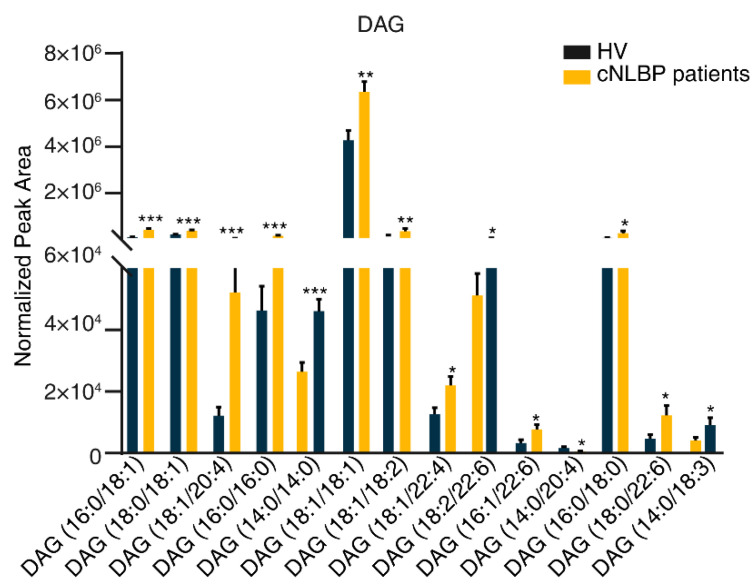
**Detecting significant variations in the constituents of DAG in the HV and cNLBP groups.** To compare the variations in DAG levels between the HV and cNLBP groups, a Student’s *t*-test was conducted. A *p*-value below 0.05 was considered statistically significant and denoted by an asterisk.

Among the 50 DAGs identified in total, 13 exhibited significantly higher abundance in the cNLBP group than in the HV group. These included DAG (16:0/18:1), DAG (18:0/18:1), DAG (18:1/20:4), DAG (16:0/16:0), DAG (14:0/14:0), DAG (18:1/18:1), DAG (18:1/18:2), DAG (18:1/22:4), DAG (18:2/22:6), DAG (16:1/22:6), DAG (16:0/18:0), DAG (18:0/22:6), and DAG (14:0/18:3). Conversely, we were surprised to find that one diacylglycerol, DAG (14:0/20:4), exhibited a significant decrease in the cNLBP group. The remaining 36 DAGs did not display significant differences between the two groups (Figure 7).

**Figure 8:**
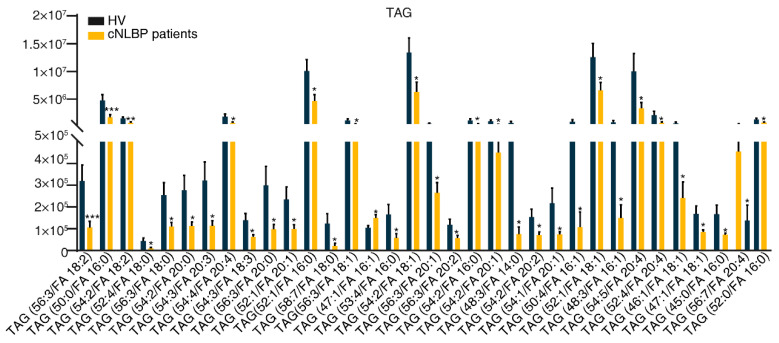
**Detecting significant variations in the constituents of TAG in the HV and cNLBP groups.** To compare the variations in TAG levels between the HV and cNLBP groups, a Student’s *t*-test was conducted. A *p*-value below 0.05 was considered statistically significant and denoted by an asterisk.

Contrary to the trends observed in MAG and DAG, our analysis of 411 TAG components revealed that approximately 10% of them exhibited a notable decrease in abundance in cNLBP patients compared to the HV group. These included TAG (56:3/FA 18:2), TAG (50:0/FA 16:0), TAG (54:2/FA 18:2), TAG (52:4/FA 18:0), TAG (56:3/FA 18:0), TAG (54:2/FA 20:0), TAG (54:3/FA 20:3), TAG (54:4/FA 20:4), TAG (54:3/FA 18:3), TAG (56:3/FA 20:0), TAG (52:1/FA 20:1), TAG (52:1/FA 16:0), TAG (58:7/FA 18:0), TAG (56:3/FA 18:1), TAG (53:4/FA 16:0), TAG (54:2/FA 18:1), TAG (56:3/FA 20:1), TAG (56:3/FA 20:2), TAG (54:2/FA 16:0), TAG (54:2/FA 20:1), TAG (48:3/FA 14:0), TAG (54:2/FA 20:2), TAG (54:1/FA 20:1), TAG (50:4/FA 16:1), TAG (52:1/FA 18:1), TAG (48:3/FA 16:1), TAG (54:5/FA 20:4), TAG (52:4/FA 20:4), TAG (46:1/FA 18:1), TAG (47:1/FA 18:1), TAG (45:0/FA 16:0), TAG (56:7/FA 20:4), and TAG (52:0/FA 16:0). Conversely, only one TAG, specifically TAG (47:1/FA 16:1), demonstrated increased abundance in the cNLBP group compared to the HV group (Figure 8).

### 3.4. Increased Levels of PS in Patients with cNLBP

**Figure 9:**
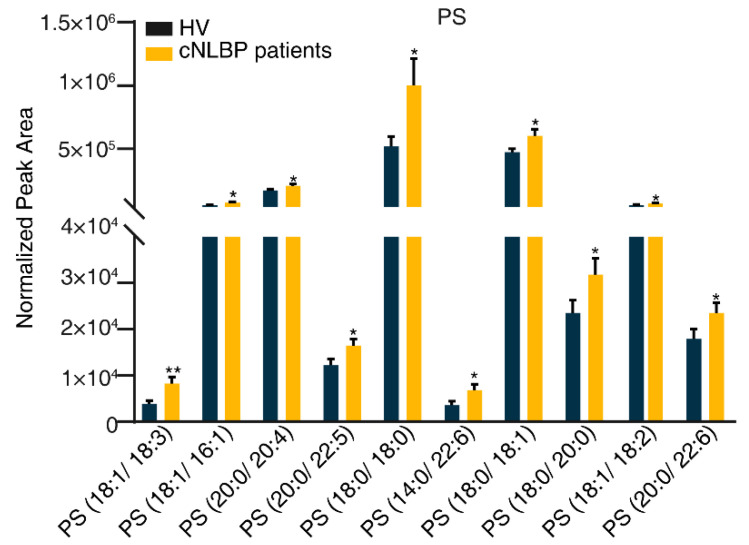
**Detecting significant variations in the constituents of PS in the HV and cNLBP groups.** To compare the variations in PS levels between the HV and cNLBP groups, a Student’s *t*-test was conducted. A *p*-value below 0.05 was considered statistically significant and denoted by an asterisk.

Among all the glycerophospholipids examined, PS was the only one that exhibited significant differences between the HV and cNLBP groups. In the cNLBP group, we observed significant increases in the abundance of 10 out of the 76 identified PSs compared to the HV group. These included PS (18:1/18:3), PS (18:1/16:1), PS (20:0/20:4), PS (20:0/22:5), PS (18:0/18:0), PS (14:0/22:6), PS (18:0/18:1), PS (18:0/20:0), PS (18:1/18:2), and PS (20:0/22:6) (Figure 9).

## 4. Discussion

cNLBP is a prevalent issue that poses challenges across various aspects of society [30]. With no clear pathogenic cause or identifiable abnormal structural factors, there is a pressing need to identify pathogenic factors linked to cNLBP. While the role of lipid signals in regulating pain sensation has been documented, it remains unclear which specific metabolites are associated with the development of cNLBP. Previous studies have primarily examined the relationship between metabolites and chronic pain conditions from the perspective of metabolomes or lipidomics [31,32,33]. However, there has been limited research employing a combined analysis of both metabolomics and lipidomics to identify biomarkers related to chronic pain. Our study identifies metabolites closely related to cNLBP through serum non-targeted metabolomics, revealing that these metabolites are primarily concentrated in the lipid metabolic pathway. Additionally, we employed serum lipidomics to pinpoint specific lipids associated with cNLBP. In summary, our study is the first to identify biomarkers of cNLBP using a combined metabolomic and lipidomic analysis, suggesting that abnormal alterations in glycerolipid levels might contribute to the development of cNLBP.

Glycerolipids are mainly synthesized in the endoplasmic reticulum (ER) using FAs and glycerol-3-phosphate substrates. PC and PE are the primary phospholipids produced through the Kennedy pathway of de novo synthesis, while PS, PI, and PG are products of the cytidine diphosphate-diacylglycerol (CDP-DAG) pathway. Within the ER, PA can also be converted into DAG, which can then undergo further acylation to form TAG [34]. Accumulating evidence points to a significant correlation between various biologically active lipids and both acute and chronic pain. One such example is the injection of 18:1-LPA, which has been demonstrated to stimulate the release of substance *p* at the end of the C-fiber nociceptor [35]. Furthermore, LPC has also been linked to inflammatory and neuropathic pain, with studies revealing that an intrathecal injection of LPC can induce strong and lasting nociceptive behaviors in mice [36]. Additionally, sphingolipids have been identified as playing a role in pain. For instance, the S1P injection has been shown to elicit spontaneous pain behaviors, such as paw flinching, which were absent in S1P receptor knockout mice [37]. However, whether cNLBP is associated with lipid composition changes remains to be established.

Our work represents one of the early efforts to examine pathogenic factors related to cNLBP from a metabolomic and lipidomic perspective. Our study uncovered a notable rise in the levels of MAG, DAG, and PS in the cNLBP group compared to the HV group. Conversely, the abundance of TAG was significantly lower in the cNLBP group, with approximately 10 percent of TAG components showing significant decreases. These data suggest that the imbalance of MAG/DAG and TAG may potentially contribute to the development of cNLBP. There exist two stereoisomers of monoacylglycerol (MAG), which are characterized by the location of the acyl chain on the glycerol framework. These include 1(3)-MAG, where the fatty acid is linked at the sn1 or sn3 position of the glycerol molecule, and 2-MAG, where the fatty acid is connected at the sn2 position [38]. Diacylglycerol (DAG) and triacylglycerol (TAG) can be converted into monoacylglycerol (MAG) through hydrolysis by diacylglycerol lipases (DAGLs) and adipose triglyceride lipases (ATGLs), respectively. Noteworthy is 2-MAG (2-arachidonoylglycerol), an endocannabinoid that provides neuroprotection and is significantly linked to pain perception [39,40]. An elevation of 2-MAG levels has been noted in preclinical models associated with inflammatory pain [41]. Several investigations suggest that administering 2-MAG topically yields analgesic outcomes [42] and that the concentrations of endogenous 2-MAG can be elevated through the application of MAGL inhibitors in various pain models [43,44,45]. These models include neuropathic pain [46], inflammatory pain [47], gastrointestinal pain [48], as well as chemotherapy-induced neuropathy [49,50].

The elevated levels of 2-MAG in patients with cNLBP may function as full agonists for CB1 and CB2 receptors [51]. The activation of these receptors in the human body typically promotes the release of neurotransmitters and plays a crucial role in pain modulation and memory learning. This may also explain the frequent occurrence of cognitive impairment in patients with cNLBP [52]. Both TAG and DAG can serve as sources of 2-MAG [53]. Consequently, a decline in TAG levels, along with an increase in DAG levels in patients with cNLBP, may lead to elevated levels of 2-MAG. This increase could potentially contribute to the progression of pain in these patients. In addition, as a crucial component of the membrane, alterations in the levels of MAG, DAG, TAG, and PS will affect membrane performance. These changes can lead to increased excitability of NMDA receptors, resulting in a corresponding rise in pain intensity among patients with cNLBP [54].

Interestingly, we observed an elevated abundance of PS in cNLBP patients compared to healthy individuals, despite previous reports linking PS to cognitive function in the brain [55]. Provision of PS was shown to improve its integration into neuronal cell membranes, which is crucial for effective neurotransmission in the human nervous system [55]. This finding aligns with our earlier observations of cognitive alterations in individuals with cNLBP [56].

The targeted analysis of lipid signals is of significant importance, as only a limited number of endogenous signaling lipids have both been identified and characterized. For instance, among the more than 250 recognized G-protein-coupled receptors (GPCRs), over 150 remain orphan receptors, many of which are likely to utilize lipids as their endogenous ligands [57]. There exists a substantial clinical demand for biomarkers related to cNLBP that can identify the disease during its initial stages, at a time when the disease’s progression may still be amenable to drug therapy. Furthermore, these biomarkers could play a vital role in personalized medicine and assist in identifying novel drug targets [58]. Recently, a variety of highly selective pharmacological tools have been developed to target the activity of enzymes that regulate specific lipid levels. These selective inhibitors are crucial for modulating pain by influencing intracellular lipid concentrations [59,60].

## 5. Conclusions and Clinical Perspective

The etiology of cNLBP remains unclear. Our study utilized metabolomic and lipidomic approaches to identify differential metabolite changes between cNLBP patients and healthy individuals. Our findings revealed significantly elevated levels of MAG, DAG, and PS in the cNLBP group compared to the HV group. In contrast, the abundance of TAG was notably lower in the cNLBP group, with a significant decrease observed in approximately 10 percent of TAG components. The opposing trends in MAG/DAG and TAG levels suggest that the imbalance of MAG/DAG and TAG could potentially impact the development of cNLBP. This study has revealed new insights into the relationship between the development of cNLBP and specific lipid components. The identification of cNLBP biomarkers facilitates the early detection of the disease, enabling timely treatment. Additionally, enzymes that regulate the levels of these specific lipids within cells may represent novel drug targets for clinical therapy.

## Figures and Tables

**Table 1:** Demographic information, mean ± SD.

	Healthy Volunteers	cNLBP Patients	*p*
N (male/total count)	11/26	9/30	
Age (years)	24.65 ± 4.49	28.50 ± 9.19	0.2806
Height (m)	1.66 ± 0.08	1.67 ± 0.11	0.8037
Weight (kg)	59.42 ± 13.52	57.00 ± 9.90	0.8074
BMI (kg/m^2^)	21.38 ± 3.50	21.87 ± 2.52	0.5523
VAS	0	4.50 ± 0.71	4.91044 × 10^−25^

## Data Availability

All data in this study can be obtained from the corresponding author up on request.

## Supplementary Materials

The following supporting information can be downloaded at https://www.mdpi.com/article/10.3390/bioengineering11111114/s1, Figure S1, Flow chart of the study.

## References

[1] Knezevic N.N., Candido K.D., Vlaeyen J.W.S., Van Zundert J., Cohen S.P. (2021). Low back pain. Lancet.

[2] Hartvigsen J., Hancock M.J., Kongsted A., Louw Q., Ferreira M.L., Genevay S., Hoy D., Karppinen J., Pransky G., Sieper J. (2018). What low back pain is and why we need to pay attention. Lancet.

[3] Hong J.Y., Song K.S., Cho J.H., Lee J.H., Kim N.H. (2022). An Updated Overview of Low Back Pain Management. Asian Spine J..

[4] Herman P.M., Coulter I.D., Hays R.D., Rodriguez A., Edelen M.O. (2022). A Scoping Review of Chronic Low Back Pain Classification Schemes Based on Patient-Reported Outcomes. Pain Physician.

[5] Dagenais S., Caro J., Haldeman S. (2008). A systematic review of low back pain cost of illness studies in the United States and internationally. Spine J..

[6] Patrick N., Emanski E., Knaub M.A. (2014). Acute and chronic low back pain. Med. Clin. N. Am..

[7] Hoy D., March L., Brooks P., Blyth F., Woolf A., Bain C., Williams G., Smith E., Vos T., Barendregt J. (2014). The global burden of low back pain: Estimates from the Global Burden of Disease 2010 study. Ann. Rheum. Dis..

[8] Xu S., Qi J., Liu C., Xia W., Wang Z., Li K., Zhou M., Liu H. (2024). Evaluation of three decades of the burden of low back pain in China before COVID-19: Estimates from the Global Burden of Disease Database 2019. J. Glob. Health.

[9] Gomez-Varela D., Barry A.M., Schmidt M. (2019). Proteome-based systems biology in chronic pain. J. Proteom..

[10] Karczewski K.J., Snyder M.P. (2018). Integrative omics for health and disease. Nat. Rev. Genet..

[11] Babu M., Snyder M. (2023). Multi-Omics Profiling for Health. Mol. Cell. Proteom. MCP.

[12] Sadee W., Wang D., Hartmann K., Toland A.E. (2023). Pharmacogenomics: Driving Personalized Medicine. Pharmacol. Rev..

[13] Teckchandani S., Nagana Gowda G.A., Raftery D., Curatolo M. (2021). Metabolomics in chronic pain research. Eur. J. Pain.

[14] Holmes E., Wilson I.D., Nicholson J.K. (2008). Metabolic phenotyping in health and disease. Cell.

[15] Zetterman T., Nieminen A.I., Markkula R., Kalso E., Lötsch J. (2024). Machine learning identifies fatigue as a key symptom of fibromyalgia reflected in tyrosine, purine, pyrimidine, and glutaminergic metabolism. Clin. Transl. Sci..

[16] Finco G., Locci E., Mura P., Massa R., Noto A., Musu M., Landoni G., d’Aloja E., De-Giorgio F., Scano P. (2016). Can Urine Metabolomics Be Helpful in Differentiating Neuropathic and Nociceptive Pain? A Proof-of-Concept Study. PLoS ONE.

[17] Livshits G., Macgregor A.J., Gieger C., Malkin I., Moayyeri A., Grallert H., Emeny R.T., Spector T., Kastenmüller G., Williams F.M.K. (2015). An omics investigation into chronic widespread musculoskeletal pain reveals epiandrosterone sulfate as a potential biomarker. Pain.

[18] Parker K.S., Crowley J.R., Stephens-Shields A.J., van Bokhoven A., Lucia M.S., Lai H.H., Andriole G.L., Hooton T.M., Mullins C., Henderson J.P. (2016). Urinary Metabolomics Identifies a Molecular Correlate of Interstitial Cystitis/Bladder Pain Syndrome in a Multidisciplinary Approach to the Study of Chronic Pelvic Pain (MAPP) Research Network Cohort. EBioMedicine.

[19] Jensen J.R., Pitcher M.H., Yuan Z.X., Ramsden C.E., Domenichiello A.F. (2018). Concentrations of oxidized linoleic acid derived lipid mediators in the amygdala and periaqueductal grey are reduced in a mouse model of chronic inflammatory pain. Prostaglandins Leukot. Essent. Fat. Acids.

[20] Ren J., Lin J., Yu L., Yan M. (2022). Lysophosphatidylcholine: Potential Target for the Treatment of Chronic Pain. Int. J. Mol. Sci..

[21] Khoury S., Colas J., Breuil V., Kosek E., Ahmed A.S., Svensson C.I., Marchand F., Deval E., Ferreira T. (2023). Identification of Lipid Biomarkers for Chronic Joint Pain Associated with Different Joint Diseases. Biomolecules.

[22] Meints S.M., Mawla I., Napadow V., Kong J., Gerber J., Chan S.T., Wasan A.D., Kaptchuk T.J., McDonnell C., Carriere J. (2019). The relationship between catastrophizing and altered pain sensitivity in patients with chronic low-back pain. Pain.

[23] van Dieën J.H., Reeves N.P., Kawchuk G., van Dillen L.R., Hodges P.W. (2019). Motor Control Changes in Low Back Pain: Divergence in Presentations and Mechanisms. J. Orthop. Sports Phys. Ther..

[24] Price D.D. (2000). Psychological and neural mechanisms of the affective dimension of pain. Science.

[25] Wang J., Zhang T., Shen X., Liu J., Zhao D., Sun Y., Wang L., Liu Y., Gong X., Liu Y. (2016). Serum metabolomics for early diagnosis of esophageal squamous cell carcinoma by UHPLC-QTOF/MS. Metabolomics.

[26] Xuan Q., Zheng F., Yu D., Ouyang Y., Zhao X., Hu C., Xu G. (2020). Rapid lipidomic profiling based on ultra-high performance liquid chromatography-mass spectrometry and its application in diabetic retinopathy. Anal. Bioanal. Chem..

[27] Xia J., Psychogios N., Young N., Wishart D.S. (2009). MetaboAnalyst: A web server for metabolomic data analysis and interpretation. Nucleic Acids Res..

[28] Xia J., Wishart D.S. (2011). Web-based inference of biological patterns, functions and pathways from metabolomic data using MetaboAnalyst. Nat. Protoc..

[29] Pang Z., Zhou G., Ewald J., Chang L., Hacariz O., Basu N., Xia J. (2022). Using MetaboAnalyst 5.0 for LC-HRMS spectra processing, multi-omics integration and covariate adjustment of global metabolomics data. Nat. Protoc..

[30] Nicol V., Verdaguer C., Daste C., Bisseriex H., Lapeyre É., Lefèvre-Colau M.M., Rannou F., Rören A., Facione J., Nguyen C. (2023). Chronic Low Back Pain: A Narrative Review of Recent International Guidelines for Diagnosis and Conservative Treatment. J. Clin. Med..

[31] Pousinis P., Gowler P.R.W., Burston J.J., Ortori C.A., Chapman V., Barrett D.A. (2020). Lipidomic identification of plasma lipids associated with pain behaviour and pathology in a mouse model of osteoarthritis. Metabolomics.

[32] Ma C., Liu M., Tian J., Zhai G., Cicuttini F., Schooneveldt Y.L., Meikle P.J., Jones G., Pan F. (2022). Lipidomic Profiling Identifies Serum Lipids Associated with Persistent Multisite Musculoskeletal Pain. Metabolites.

[33] Gonzalez P.A., Simcox J., Raff H., Wade G., Von Bank H., Weisman S., Hainsworth K. (2022). Lipid signatures of chronic pain in female adolescents with and without obesity. Lipids Health Dis..

[34] Farese R.V., Walther T.C. (2023). Glycerolipid Synthesis and Lipid Droplet Formation in the Endoplasmic Reticulum. Cold Spring Harb. Perspect. Biol..

[35] Renbäck K., Inoue M., Yoshida A., Nyberg F., Ueda H. (2000). Vzg-1/lysophosphatidic acid-receptor involved in peripheral pain transmission. Brain Res. Mol. Brain Res..

[36] Inoue M., Xie W., Matsushita Y., Chun J., Aoki J., Ueda H. (2008). Lysophosphatidylcholine induces neuropathic pain through an action of autotaxin to generate lysophosphatidic acid. Neuroscience.

[37] Camprubí-Robles M., Mair N., Andratsch M., Benetti C., Beroukas D., Rukwied R., Langeslag M., Proia R.L., Schmelz M., Ferrer Montiel A.V. (2013). Sphingosine-1-phosphate-induced nociceptor excitation and ongoing pain behavior in mice and humans is largely mediated by S1P3 receptor. J. Neurosci. Off. J. Soc. Neurosci..

[38] Poursharifi P., Madiraju S.R.M., Prentki M. (2017). Monoacylglycerol signalling and ABHD6 in health and disease. Diabetes Obes. Metab..

[39] Calignano A., La Rana G., Giuffrida A., Piomelli D. (1998). Control of pain initiation by endogenous cannabinoids. Nature.

[40] Deng H., Li W. (2020). Monoacylglycerol lipase inhibitors: Modulators for lipid metabolism in cancer malignancy, neurological and metabolic disorders. Acta Pharm. Sin. B.

[41] Beaulieu P., Bisogno T., Punwar S., Farquhar-Smith W.P., Ambrosino G., Di Marzo V., Rice A.S. (2000). Role of the endogenous cannabinoid system in the formalin test of persistent pain in the rat. Eur. J. Pharmacol..

[42] Guindon J., Desroches J., Beaulieu P. (2007). The antinociceptive effects of intraplantar injections of 2-arachidonoyl glycerol are mediated by cannabinoid CB2 receptors. Br. J. Pharmacol..

[43] Khasabova I.A., Chandiramani A., Harding-Rose C., Simone D.A., Seybold V.S. (2011). Increasing 2-arachidonoyl glycerol signaling in the periphery attenuates mechanical hyperalgesia in a model of bone cancer pain. Pharmacol. Res..

[44] Guindon J., Guijarro A., Piomelli D., Hohmann A.G. (2011). Peripheral antinociceptive effects of inhibitors of monoacylglycerol lipase in a rat model of inflammatory pain. Br. J. Pharmacol..

[45] Hohmann A.G., Suplita R.L., Bolton N.M., Neely M.H., Fegley D., Mangieri R., Krey J.F., Walker J.M., Holmes P.V., Crystal J.D. (2005). An endocannabinoid mechanism for stress-induced analgesia. Nature.

[46] Guindon J., Lai Y., Takacs S.M., Bradshaw H.B., Hohmann A.G. (2013). Alterations in endocannabinoid tone following chemotherapy-induced peripheral neuropathy: Effects of endocannabinoid deactivation inhibitors targeting fatty-acid amide hydrolase and monoacylglycerol lipase in comparison to reference analgesics following cisplatin treatment. Pharmacol. Res..

[47] Ghosh S., Wise L.E., Chen Y., Gujjar R., Mahadevan A., Cravatt B.F., Lichtman A.H. (2013). The monoacylglycerol lipase inhibitor JZL184 suppresses inflammatory pain in the mouse carrageenan model. Life Sci..

[48] Kinsey S.G., Nomura D.K., O’Neal S.T., Long J.Z., Mahadevan A., Cravatt B.F., Grider J.R., Lichtman A.H. (2011). Inhibition of monoacylglycerol lipase attenuates nonsteroidal anti-inflammatory drug-induced gastric hemorrhages in mice. J. Pharmacol. Exp. Ther..

[49] Kinsey S.G., Long J.Z., Cravatt B.F., Lichtman A.H. (2010). Fatty acid amide hydrolase and monoacylglycerol lipase inhibitors produce anti-allodynic effects in mice through distinct cannabinoid receptor mechanisms. J. Pain.

[50] Kinsey S.G., Long J.Z., O’Neal S.T., Abdullah R.A., Poklis J.L., Boger D.L., Cravatt B.F., Lichtman A.H. (2009). Blockade of endocannabinoid-degrading enzymes attenuates neuropathic pain. J. Pharmacol. Exp. Ther..

[51] Sugiura T., Kondo S., Kishimoto S., Miyashita T., Nakane S., Kodaka T., Suhara Y., Takayama H., Waku K. (2000). Evidence that 2-arachidonoylglycerol but not N-palmitoylethanolamine or anandamide is the physiological ligand for the cannabinoid CB2 receptor. Comparison of the agonistic activities of various cannabinoid receptor ligands in HL-60 cells. J. Biol. Chem..

[52] Abd-Elsayed A., Gyorfi M. (2023). Chronic low back pain and cognitive function. Pain Pract. Off. J. World Inst. Pain.

[53] Loo L., Wright B.D., Zylka M.J. (2015). Lipid kinases as therapeutic targets for chronic pain. Pain.

[54] Mifflin K.A., Kerr B.J. (2014). The transition from acute to chronic pain: Understanding how different biological systems interact. Can. J. Anaesth..

[55] Glade M.J., Smith K. (2015). Phosphatidylserine and the human brain. Nutrition.

[56] Zeng X., Tang W., Gao F., Tang Z., Zhang Z., Zhang J., Du M., Chen Z., Chen X., Yuan Z. (2023). Behavioral modeling and neuroimaging of impaired risky decision making in patients with chronic musculoskeletal pain. Neurophotonics.

[57] Kroeze W.K., Sheffler D.J., Roth B.L. (2003). G-protein-coupled receptors at a glance. J. Cell Sci..

[58] Mobasheri A., Henrotin Y. (2015). Biomarkers of (osteo)arthritis. Biomarkers.

[59] Baggelaar M.P., Chameau P.J., Kantae V., Hummel J., Hsu K.L., Janssen F., van der Wel T., Soethoudt M., Deng H., den Dulk H. (2015). Highly Selective, Reversible Inhibitor Identified by Comparative Chemoproteomics Modulates Diacylglycerol Lipase Activity in Neurons. J. Am. Chem. Soc..

[60] Ogasawara D., Deng H., Viader A., Baggelaar M.P., Breman A., den Dulk H., van den Nieuwendijk A.M., Soethoudt M., van der Wel T., Zhou J. (2016). Rapid and profound rewiring of brain lipid signaling networks by acute diacylglycerol lipase inhibition. Proc. Natl. Acad. Sci. USA.

