# Supplementary figures and images for: Lipid Alterations in Chronic Nonspecific Low Back Pain in the Chinese Population: A Metabolomic and Lipidomic Study
Source: Bioengineering (Basel). 2024 Nov 5;11(11):1114. doi: 10.3390/bioengineering11111114 (PMC11591451; doi:10.3390/bioengineering11111114)

Supplemental Figure S1. Flow chart of the study

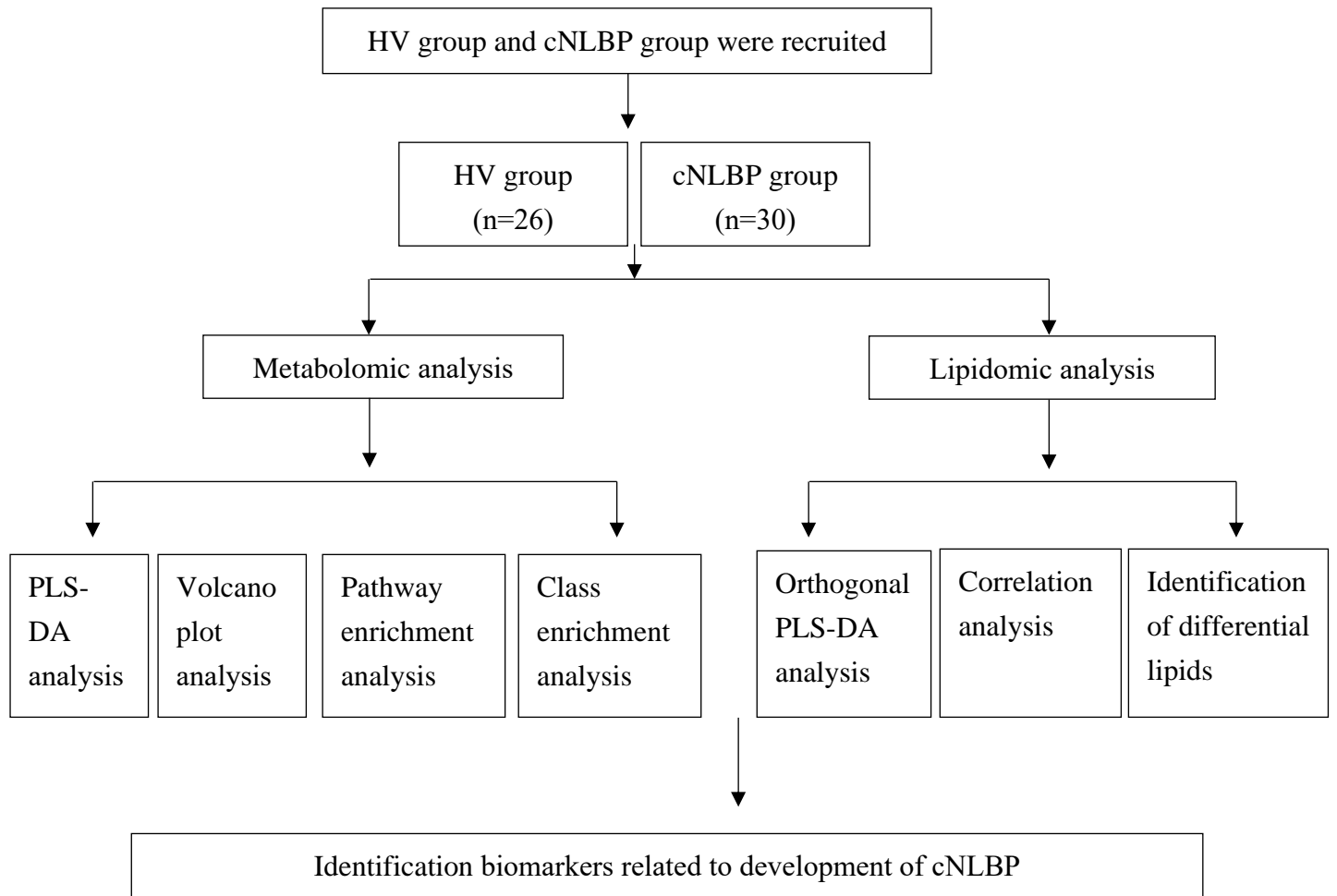

Supplement: Supplementary file 1 [file bioengineering-11-01114-s001.zip › bioengineering-3256256-supplementary.pdf]
